# Digital interventions for common mental health problems among older adults in low- and middle-income countries: a scoping review

**DOI:** 10.1136/bmjgh-2024-017836

**Published:** 2025-06-24

**Authors:** Indranil Saha, Christopher Sundström, Arun Kandasamy, Martin Kraepelien, Neha Dahiya, Asim Saha, Nitya Jayaram-Lindström, Amit Chakrabarti, Vivek Benegal

**Affiliations:** 1 Centre for Ageing and Mental Health, Indian Council of Medical Research, Kolkata, West Bengal, India; 2 Centre for Psychiatry Research, Department of Clinical Neuroscience, Karolinska Institute, Stockholm, Sweden; 3 Psychiatry, National Institute of Mental Health and Neuro Sciences, Bengaluru, Karnataka, India; 4 ICMR Headquarter, Indian Council of Medical Research, New Delhi, India

**Keywords:** Aging, Review, Internet, Global Health, Mental Health & Psychiatry

## Abstract

**Background:**

The proportion of older adults is projected to rise dramatically in most parts of the world, and in 2050, 80% of all older adults (≥65 years) will reside in low- and middle-income countries. Developing digital interventions tailored to this population will be a crucial part in appropriately addressing the challenges associated with the upcoming demographic changes. The objective of this scoping review was to map all published research on digital interventions for common mental health problems among older adults conducted in low- and middle-income countries.

**Methods:**

We conducted a literature search in four databases (PubMed, Embase, Scopus and Cochrane Library) and conducted hand searches of existing reviews and reference lists of included studies. Eligibility criteria were that (1) the study investigated or discussed digital interventions for common mental health problems (depression, anxiety, sleep problems and/or substance use problems), (2) the study concerned older adults (at least 60 years or older), (3) the study was conducted in a low- or middle-income country and (4) the study was published in English in a peer-reviewed academic journal. Two reviewers independently screened, reviewed and extracted data.

**Results:**

Out of 1344 identified studies, 6 were included. The hand searches rendered no additional studies, leaving a total of six included studies. Four of these studies included quantitative data: of these, three were randomised controlled trials and one was a feasibility study. All focused on depression/loneliness and were published in middle-income countries (South Africa, Brazil and Turkey), and showed promising results in terms of reductions in social isolation and depression. In addition, two expert opinions had been published, both also conducted in a middle-income country (India).

**Conclusion:**

Despite the projected demographic transition, surprisingly little research appears to have been conducted on digital interventions for common mental health disorders among older adults in low- and middle-income countries.

**Registration:**

https://osf.io/bmdnv Open Science Framework.

**WHAT IS ALREADY KNOWN ON THIS TOPIC**

- The global population of older adults is rapidly increasing, especially in low- and middle-income countries (LMICs) where 80% of older adults will reside by 2050. Despite this demographic shift, there appears to have been limited research on digital interventions for mental health problems tailored to this group in LMICs.

**WHAT THIS STUDY ADDS**

- This scoping review identified only six studies related to digital interventions for mental health problems among older adults in LMICs. Randomised controlled trials have been conducted in middle-income countries (South Africa, Brazil, Turkey) primarily targeting depression and loneliness with promising results in reducing social isolation and other mental health symptoms.

**HOW THIS STUDY MIGHT AFFECT RESEARCH, PRACTICE OR POLICY**

- The findings suggest an urgent need to scale up research on digital mental health interventions in LMICs to address the growing mental health needs of older adults. If found effective, such interventions can be used as part of comprehensive care for mental health in ageing populations.

## Introduction

 Following medical advancements and sinking fertility rates,^1 2^ the proportion of older adults (≤65 years) in the world is projected to increase in the coming decades, from a figure of 12% in 2015 to an estimated 22% in 2050.^3^ In high-income countries (HICs) in Europe, 20% of the population are currently 65 or over,^4^ which represents a doubling in the past 50 years (1972: 11.8%, 2022: 21.2%).^5^ In Japan, the most extreme example in terms of proportions, almost 30% are currently over the age of 65, and 10% are 80 years or older.^5 6^ Many low- and middle-income countries (LMICs) also display proportional increases. In India, the percentage of older adults has doubled in the past 50 years (from 3.7% to 6.9%), in China it has almost quadrupled (from 3.8% to 13.7%),^5 7^ and projections are that these percentages will increase even further in the coming decades. Although proportions are higher in HICs, the number of older adults in society is much greater in LMICs,^8 9^ with recent estimates holding that almost 70% of the world’s older population resides in these countries.^10^ In 2050, this figure is projected to be 80%.^11^ This is particularly alarming given the fact that many LMICs do not have developed healthcare infrastructure for older adults.^8 12 13^ Doubtlessly, this ongoing development signifies a monumental demographic shift leading to substantial challenges for society, economy and healthcare in these countries,^14^,^16^ and one important aspect to successfully address the shift will be to develop, and ensure equitable access to, preventive healthcare interventions for older adults.^17^

Older adults have multiple and inter-related challenges. Aside from age-related functional decline and somatic diseases, mental health problems are common among older adults, both in HICs^18 19^ and in LMICs.^20^ Problems such as loneliness and social isolation^21^,^23^ anxiety,^24^ insomnia^25 26^ and substance use^27^ are prevalent. More than for other age groups, mental and somatic health among older adults are intrinsically entwined, as shown, for example, when depression is found to predict cognitive decline,^28^ when loneliness is identified as a risk factor for mortality,^29^ or when anxiety increases the risk of coronary artery disease.^30^ Many older adults are medically treated for somatic and psychiatric conditions simultaneously, leading them to suffer negative consequences of polypharmacy, such as adverse drug reactions, falls, low compliance and persistence of symptoms.^31 32^ At the same time, non-pharmacological interventions for older adults are highly underused.^33^ In many LMICs, informal caregiving and care of older adults provided by family members is the norm,^34^ often leading to high caregiver burden.^35 36^ Access to mental health services (ie, psychiatrists, psychologists, mental health nurses) is scarce,^37 38^ despite the major impact of mental health conditions on disability among older adults.^3^ There is thus a clear and urgent need to develop innovative non-pharmacological ways to prevent and treat mental health problems among older adults in LMICs,^8 39^ and designing such interventions has therefore been deemed a top research priority.^40^

In 2023, 65% of the world had access to the internet.^41^ In HICs, 93% of the population had such access, in middle-income countries the number was 67%, and in low-income countries, it was 27%.^42^ Internet penetration is bound to increase even more in LMICs in the coming years, and new cohorts of older adults in these countries are sure to be more frequent internet users than previous cohorts. Digital interventions have immense potential to address mental health issues among older adults, for example, by remotely delivering cognitive–behavioural therapy (CBT) and psychoeducation as well as general strategies related to healthy ageing.^43^ Digital interventions for mental health problems could be especially beneficial for older adults. First, they do not require visits to healthcare facilities, thereby reducing common age-related treatment barriers such as cost, transportation and mobility. Second, the scalability of digital mental health interventions means that they could significantly increase accessibility to much-needed prevention for older adults in an equitable way.^17^ Third, if effective, these interventions could possibly decrease prescriptions of antidepressants, reducing the inherent risks of polypharmacy for older adults.^32^ Fourth, digital interventions are more anonymous than face-to-face treatment, which may circumvent the self-stigma related to mental health^44^ suggested to be more prevalent among older adults.^45 46^ Several systematic reviews show that digital interventions for mental health problems among older adults can effectively reduce symptoms of depression and anxiety, with small to moderate effect sizes.^47^,^53^ However, none of these reviews focused on LMICs. Conversely, several systematic reviews on digital interventions for mental health problems in LMICs have been published showing that these interventions are effective in treating depression and anxiety, but none of these reviews had a specific focus on older adults.^54^,^57^ It is thus unclear to what extent research on digital interventions for older adults in LMICs has been conducted. A scoping review, encompassing the extent, range and nature of research activity conducted in this area could contribute to understanding its current state as well as its research gaps.

In the current study, we conducted a scoping review to answer the question: what do we know from the existing literature about digital interventions for common mental health problems among older adults in LMICs?

## Methods

### Study design

The study was a scoping review attempting to summarise all published literature on digital interventions for common mental health problems among older adults in LMICs. Databases were searched to address the research question in PICO format. The protocol was registered in Open Science Framework platform (https://doi.org/10.17605/OSF.IO/BMDNV). All reporting was conducted in accordance with Arksey and O’Malley stages of framework^58^ and with the Preferred Reporting Items for Systematic Reviews and Meta-Analyses Extension for Scoping Reviews (PRISMA-ScR).^59^ See Preferred Reporting Items for Systematic Reviews and Meta-Analyses checklist (online supplemental appendix 1).

### Search strategy and selection criteria

First, the research question was broken down into different components as per P (Population/patient), I (Intervention), C (Comparator), O (Outcome) and S (Settings) format. However, as this was a scoping review and not a systematic review, the O (Outcome) was excluded (see table 1 for search details). From the different components, concepts were identified, followed by identification of related MeSH term and tiab (*ti*tle *ab*stract) term (online supplemental appendix 2). Searches with the identified MeSH terms and tiab terms were conducted on the four databases PubMed, Embase, Scopus and Cochrane Library (search strategy in Scopus database is outlined in online supplemental appendix 3). Further, reference lists of included studies and previously published reviews on digital interventions for mental health problems among older adults were hand-searched to identify additional eligible studies.

**Table 1:** Summary of included studies with quantitative data

References, country	Year	Setting and recruitment	Study design	Mental health focus	Intervention name	Intervention content and delivery	Duration of intervention	Inclusion criteria	n (% female)	Age (m, SD)	Mental health outcomes	Findings
Jarvis *et al*, South Africa	2019	Non-governmental organisation offering accommodation for resource-restricted residents	RCT with two groups: Intervention group (n=15) Control group (generic wellness programme) (n=17)	Loneliness	Living in Networked Communities	Intervention consists of four phases: (1) technology acceptance through selection of smartphone and four 90-minute group sessions (2 weeks), (2) psychoeducation about loneliness delivered over 90-minute face-to-face sessions as well as over WhatsApp (2 weeks), (3) and individualised messages aimed at maladaptive cognitions sent over WhatsApp (1 month), (4) maintenance stage (1 month)	12 weeks	Participants must: Be 60 years or over Reside in the residence Be willing to participate Be cognitively intact In addition to the above, the participant must also either Be socially isolated Experience loneliness Have decreased mental well-being	26 (81.3)	74.9 (6.4)	Social cognition: Young Schema Questionnaire (YSQ) Loneliness: De Jong Gierveld Loneliness Scale (DGLS) Mental well-being: WHO-5	There were significant positive changes in both YSQ and DGLS At 1-month follow-up, a significant reduction in DGLS was maintained
Tekin and Cetisli-Korkmaz, Turkey	2022	Grandparents of physiotherapy interns	RCT with two groups: Intervention group No formal exercise training	Depression and fall prevention	No name	Home exercise programme consisting of six different types of calisthenic exercises. Videos of exercise prescriptions were sent to participants via online methods. The exercise programme was scheduled 5 days a week for 4 weeks	4 weeks	Participants must: Volunteer Be 65 years or over Not have serious cardiac, orthopaedic or cognitive problems	255 (45.9)	70.3 (5.4)	Geriatric Depression Scale (GDS) Modified Fall Efficacy Scale (MFES) Short Physical Performance Battery (SPPB)	Significant improvements in GDS and MFES in the intervention group
Brandão *et al*, Brazil	2022	University hospital/public community healthcare centre etc	Feasibility study with a mixed-methods approach	Social isolation	Playful Living	Weekly 60-minute WhatsApp video calls with undergraduate students with knowledge of four areas of arts and health (clowning, dancing, storytelling, cooking)	12 weeks	Participants must: Be 60 years or older Have low SES Have a history of stroke or dementia Have no neurological diseases Experience loneliness Live with a relative or someone who can help with technical issues	34 (58.3)	71.8 (9.2)	Quantitative: Depression Geriatric Depression Scale (GDS) Geriatric Anxiety Inventory (GAI) were used at screening Qualitative: Participant observation and thematic analysis (TA)	Depression and anxiety appeared less common after treatment, although loss to follow-up was high. TA showed that participants perceived the programme as supportive and reported feelings of belonging. No negative consequences were reported in relation to the experience during or after one‐to‐one or group calls
Scazufca *et al*, Brazil	2024	Primary care	RCT with two groups: Intervention group Control group (participant receiving only one message)	Depression	Viva Vida	Intervention consists of 48 audio and visual messages, delivered 4 days a week in the morning and afternoon over a 6-week period on WhatsApp. Content-wise, the intervention consists of psychoeducation about depression, health promotion guidelines, simple ways to solve day-to-day problems related to depressive symptoms and behavioural activation	6 weeks	The participant must: Be 60 years or over Be registered with any of the participating primary care clinics (socioeconomically disadvantaged area) Be able to receive and listen to WhatsApp messages Have depressive symptomatology	603 (74.8)	65.1	Patient Health Questionnaire-9 (PHQ-9)	Significantly more had improved depressive symptomatology according to the PHQ-9

RCTs, randomised controlled trials; SES, socioeconomic status.

All retrieved articles from the four different databases were entered into Rayyan software (https://www.rayyan.ai/). First, duplicate articles were identified and deleted. After duplicate removal, the title and abstract of the remaining articles were reviewed independently by two authors (IS and CS). After that, a full text screening followed where all relevant articles were downloaded and scrutinised to determine eligibility. In each stage, any discrepancies that arose during title and abstract screening as well as during the full text screening between the two authors (IS and CS) were resolved through consensus. Data from the studies were then charted in a data extraction form under the headings of key variables related to stated objectives^58^; countries, study designs, interventions and outcomes. The following selection criteria were employed:

1. The study investigated or discussed digital interventions for common mental health problems (depression, anxiety, sleep problems and/or substance use problems).
2. The study concerned older adults (at least 60 years or older).
3. The study was conducted in an LMIC as defined by the World Bank in the fiscal year of 2023.^60^
4. The study was published in English in a peer-reviewed academic journal.

As this was a scoping review attempting to include all published research in the area, we included the following types of studies: randomised controlled trials (RCTs), non-RCTs, uncontrolled trials, observational analytical studies of prospective or retrospective design, case studies, case series, expert opinions, narrative reviews, editorials, commentary and conference proceedings.

### Patient and public involvement

Neither patients nor public were involved in the design, conduct, reporting or dissemination plans of this scoping review.

## Results

**Figure 1:**
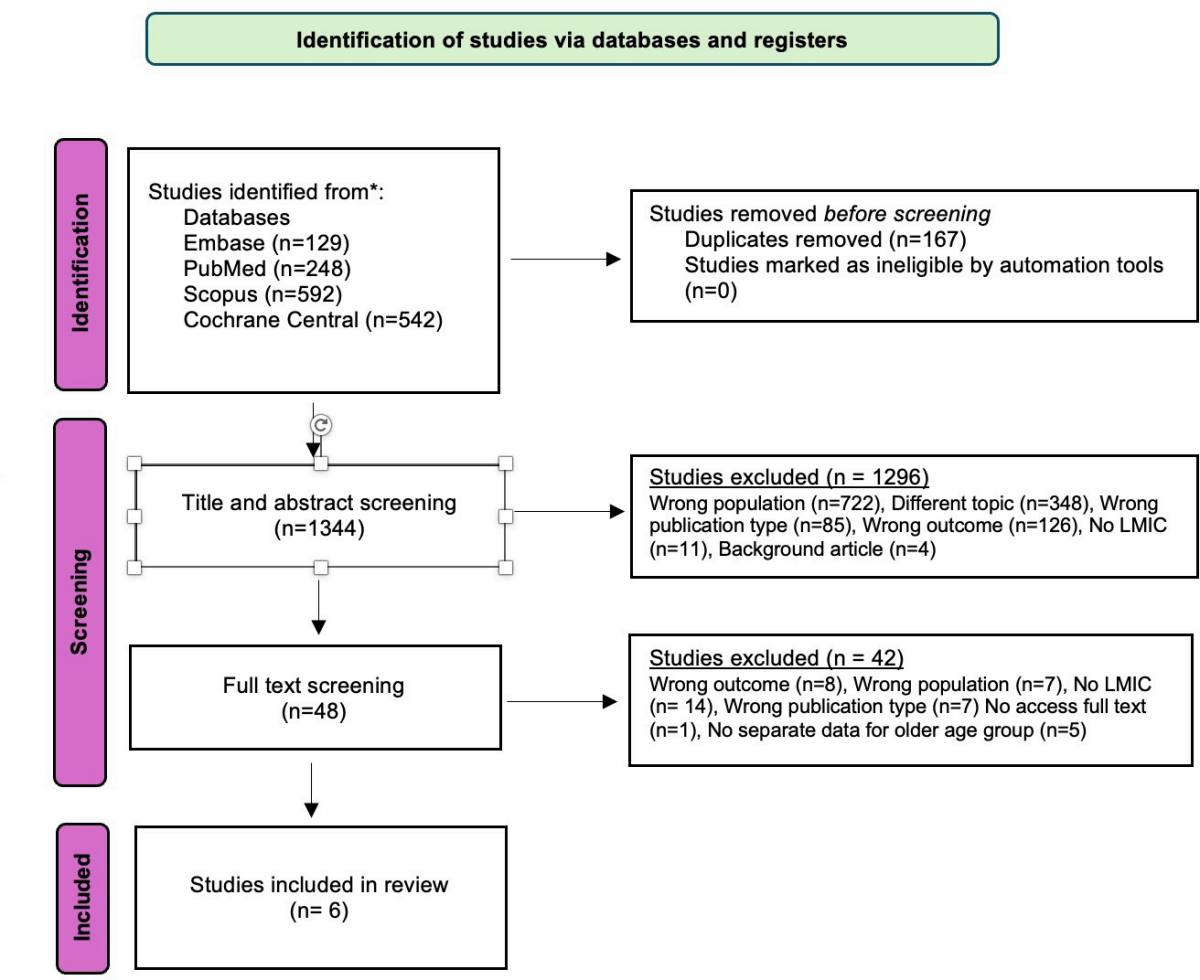
Preferred Reporting Items for Systematic Reviews and Meta-Analyses 2020 flow diagram. LMIC, low- and middle-income country.

The literature search was conducted on 17 April 2024, and yielded 1511 articles. Before screening, 167 duplicate articles were removed. Title and abstract of the remaining 1344 articles were then screened, which was followed by a full-text screening of 48 articles. After the full text screening, six articles were included. The reason for the exclusion of different articles in the different stages is reported in accordance with PRISMA-ScR.^59^ After the literature search, a hand search of previously published reviews on digital interventions for older adults, as well as of reference lists in the six included studies from the literature search (backward search) was conducted, which did not lead to any additional articles identified. Thus, in all, six articles were included, four of which included quantitative data and two of which were viewpoint articles. See figure 1 for a flow chart and see table 1 for a presentation of the four included studies presenting quantitative data.

### Data extraction from included studies with quantitative data

#### Geographic and temporal distribution of studies

Of the four studies, all were from middle-income countries; two were from Brazil, one was from Turkey and one was from South Africa. No studies were thus from low-income countries. All articles had been published in the last 5 years (2019–2024).

#### Study designs, recruitment, inclusion criteria, control groups and sample size

Jarvis *et al*^61^ conducted an RCT in a large city in South Africa where older adults residing in accommodation provided by a non-governmental organisation were randomised to either the *Living in Networked Communities* intervention, or to a control group which consisted of a general wellness programme. Included were those 60 years or over who were cognitively intact and who reported social isolation on the Friendship Scale, loneliness on the de Jong Gierveld Loneliness Scale and/or decreased mental well-being on the WHO-Five Well-being Scale. In all, 32 individuals were included, which was lower than the estimated needed sample size of 52. Scazufca *et al*^62 63^ conducted an RCT in Guarulhos, a large city in Brazil, where older adults (≤60 years) reporting symptoms of depression on the Patient Health Questionnaire-9 were recruited through primary care clinics and randomised to either the *Viva Vida* intervention or to a control arm receiving a single message with information about depression. In all, 603 individuals were included. Tekin and Cetisli-Korkmaz^64^ conducted an RCT in Turkey where grandparents of physiotherapy interns who were older adults (≤65 years) and without serious cardiac, orthopaedic or cognitive problems, were randomised to a physical calisthenic exercise programme consisting of video sessions distributed online, or to a control group asked to only complete the assessments and not participate in any structured exercise programme during the study period. No inclusion criteria based on depression or other mental health problems were applied in the trial. In all, 255 participants were included. Finally, Brandão *et al*^65^ conducted a feasibility mixed-methods study in the south of Brazil where older adults (≥60 years) who were socioeconomically vulnerable and had stroke-induced cognitive impairments, dementia or aphasia, expressing experiencing loneliness according to social service and having a relative or social educator who could be of assistance, were offered the programme *Playful Living*, designed to promote well-being and social connection. The researchers used convenience sampling and aimed to reach diverse ethnic backgrounds. Here, 34 individuals were included. As this was a feasibility study, no control group was included.

#### Interventions

In Jarvis *et al*, the intervention was informed by a theoretical model of loneliness and CBT and specifically aimed at changing maladaptive cognitions. It consisted of four phases spaced out over 2 months; phase 1 (2 weeks) consisted of smartphone selection and three 90-minute group sessions on technology acceptance, phase 2 (2 weeks) consisted of four 90-minute face-to-face sessions on psychoeducation about loneliness, phase 3 (1 month) consisted of WhatsApp messages aimed at maladaptive cognitions and phase 4 was a maintenance stage. In Scazufca *et al*, the intervention consisted of audio and visual messages delivered via WhatsApp and was based on CBT. The messages delivered contained psychoeducation about depression and health promotion guidelines, problem-solving and behavioural activation. A total of 48 messages were delivered 4 days a week over 6 weeks. In Tekin and Cetisli-Korkmaz, the programme contained descriptions of six different types of calisthenic exercises (Speed Jack, Shoulder Bridge, Superman, Lunge, Strong-Man Flexion and Trunk Rotation) that have been shown to decrease both falling risk and depression. These exercises were scheduled for the participants 5 days a week for 4 consecutive weeks. Participants were instructed to record themselves digitally while performing the exercises and send the videos to the researchers (who were physiotherapists), who then reviewed the videos and informed participants if there were problems observed in their exercise practices. Participants in the control group were asked not to participate in any exercise programme during the study but to only fill out the two assessments. In Brandão *et al*, the intervention consisted of both one-to-one regular WhatsApp video calls each week with an undergraduate student as well as streaming group sessions focusing on either of four specific artistic activities (dancing, clowning, storytelling or cooking). The streaming group session consisted of 10 min welcome, 10 min warm-up, 20 min devoted to the main activity (dancing, clowning, storytelling and cooking) and 10 min farewell. The intervention did not appear to be based on any specific theory.

#### Measures and outcomes

In Jarvis *et al*, outcome measures were social cognition, loneliness and mental well-being. There were significant positive changes in both loneliness and social cognition at first follow-up, and at the 1-month follow-up, the significant effect on loneliness was maintained. In Scazufca, at the 3-month follow-up, the active intervention showed a significantly higher increase in odds of improvement from depression compared with the control group. However, at the 5-month follow-up, no such differences were observed. In Tekin and Cetisli-Korkmaz, at the 4-week follow-up, there were significant differences between the groups in that there were lower levels of depression (using the Geriatric Depression Scale; GDS) and fear of falling in the intervention group, as well as higher levels of standing and balance skills, when compared with the control group. In Brandão *et al*, the programme was generally appreciated according to thematic analyses of evaluative conversations. Fewer participants appeared to suffer from depression at follow-up, but no statistical calculations of to what extent measures of depression or anxiety had changed were presented.

#### Human interaction

In Jarvis *et al*, the intervention consisted of four phases, with the first phase being three 90-minute group sessions on technology acceptance, the second phase consisting of four 90-minute face-to-face sessions on psychoeducation about loneliness and the last two phases dedicated to digitally delivered content. In Scazufca *et al*, no human support aside from technical support was included in the study. In Tekin and Cetisli-Korkmaz, telephone contact was made only with those older adults who had problems in their exercise practices, while Brandão *et al* included extensive video calls with both undergraduate students and with groups.

### Data extraction from the two included expert opinion articles

The two expert opinion articles both discussed the implementation of digital interventions for older adults in Bengaluru, India. Sivakumar *et al*^66^ described work conducted at the Telemedicine Unit, National Institute of Mental Health Services in Bengaluru. Here, video consultations have been successfully used as follow-up care with psychiatry patients, including geriatric psychiatry patients, since 2017. An advantage of assessments made through audio or video mode from the patient’s home lifted by the authors was that these assessments provided clinicians with the natural environment of patients. It was also noted by the authors that the presence of family caregivers facilitated the implementation of telemedicine. Further, a group intervention developed for dementia caregivers had been provided through video conferencing and had been successful among caregivers who otherwise had difficulties attending. Further, Kalaivanan *et al*^67^ assessed opportunities and challenges of providing digital interventions to older adults in India. Facilitators mentioned by the authors were a lack of social support from family members, restrictions of mobility due to old age, severity of symptoms of other diseases and financial constraints. Clinically stable patients with no or low cognitive impairment, milder forms of mental illness, as well as patients from higher socioeconomic status, generally appeared more comfortable with digital intervention services. Poor internet connectivity, software-related problems like difficulty in installation, difficulty in navigation of the application and difficulty in troubleshooting were mentioned as barriers in implementing digital interventions.

## Discussion

### Synthesis of findings

In the present scoping review, we attempted to identify all published research on digital interventions for common mental health problems among older adults conducted in LMICs. Only six studies were identified. Four of these studies reported some form of quantitative data collection; three being RCTs and one being a feasibility study. All focused primarily on depression and social isolation (no studies thus focused on other mental health problems prevalent in this age group, such as sleep problems, anxiety and substance use). Recruitment differed among the studies and inclusion criteria were in two studies based partly on validated questionnaires.^61 62^ Sample sizes were several hundred in two cases^62 64^ and around 30 in the two other cases.^61 65^ Only Brandão *et al* included older adults with dementia and aphasia,^65^ in other studies, such conditions were excluded. Regarding therapeutic content, two of the included studies investigated interventions based on CBT,^61 62^ one focused on calisthenic exercise and one focused on encouraging playful activities. None of the studies used a specific platform developed for the intervention. Instead, messaging services and video calls were used to deliver the content. The extent of human interaction differed between the studies; in two studies, human interaction was quite extensive,^61 65^ while in the two other studies, human interaction was quite restrictive.^62 64^ All four studies reported promising results in terms of reducing depression and social isolation, although follow-up times were admittedly short. It should be noted that a psychiatric diagnosis was not required in any of the studies, so generalisations cannot be made to clinical populations. Geographically, all studies were conducted in middle-income countries. Surprisingly, none of the four studies with quantitative data had been conducted in Asia which is surprising since this geographical area harbours the majority of older adults in the world. The finding that all studies had been published in the past 5 years (2019–2024), indicates that this is a new and growing field. As for the two expert opinion articles, both discussed the implementation of geriatric telepsychiatry services in India and its facilitators and barriers. These articles indicate that implementation efforts may precede scientific publishing, at least at a regional level.

### Strengths and limitations

There are several strengths to this scoping review. To the best of our knowledge, this is the first review focusing on digital interventions for mental health problems among older adults in LMICs and so our results are an important step in expanding research in this area. Second, we conducted a full and thorough search on four different databases, as well as conducted hand searches on previously published reviews on digital interventions for mental health problems among older adults and reviews on digital interventions for mental health problems in LMICs. However, some limitations should be considered when interpreting the findings. First, although this review did employ a comprehensive search strategy, conducting searches across four widely used databases, it is possible that some studies were not identified in the searches of these particular databases. We did not conduct searches in PsycINFO, and although there is substantial overlap in terms of indexed journals between this database and the ones we used in our searches, this should be noted as a limitation. Further, there are databases important in some LMIC regions, for example, South America (Scielo and LILACS) and China (CNKI, Wanfang), that we did not use. Also, our searches only included studies published in English. Although the databases that we used include studies in other languages, providing them with an English name, we cannot rule out that there may have been studies published in languages other than English not identified by our searches. It should also be noted that grey literature was not included in the search, and so there may be studies conducted in non-research settings that could nuance our findings. Second, we did not identify any studies conducted in low-income countries, so the results from the current review may only be generalised to middle-income countries (to the extent they can be generalised at all). A previous review looking at digital interventions for common mental disorders in LMICs did not find any studies at all conducted in low-income countries either.^57^ As noted in that review, this lack of studies may have varying explanations, mostly related to the digital divide (ie, limited access to the internet and mobile phones in these countries). Internet penetration is indeed lower in low-income countries compared with middle-income countries.^42^ An increasing global internet penetration, however, will enable studies investigating digital interventions for older adults and other populations in these countries, and only then will we know whether these interventions may be feasible there. Third, we only included studies on digital interventions focusing on mental health specifically. One previous review on digital interventions for older adults included studies such as memory training and rehabilitation for Parkinson’s disease.^53^ Although the case can be made for such inclusion, we decided not to include these studies. This also means that we did not include studies solely focusing on healthy ageing. Fourth, we used the age of 60 as an inclusion criterion, whereas other reviews on digital interventions for older adults have used 50 or older as an exclusion criterion instead.^47 53^ Fifth and finally, criticism has been directed towards the overuse of the term LMICs, suggesting that the categorisation can obscure both commonalities and differences across countries.^68^ LMICs is an umbrella term used by the World Bank to signify the more than 130 countries with a particular gross national income, but obviously these countries are highly diverse in terms of both cultures, languages and healthcare service infrastructure. Although we hope that our review can contribute to providing an overview of published research so as to better assess the needs of these countries, we do acknowledge the limitations of this categorisation and agree that the term should not be used frivolously.

### What is needed for the field to advance?

From our scoping review, we can conclude that digital interventions appear feasible among older adults in LMICs. However, research is still at an early stage and there is much that could be done to advance the field. Future research could focus on a number of aspects. First, involving family members is crucial and may be even more pivotal when developing digital interventions for older adults in LMICs, as the infrastructure of health services for older adults in these countries often is insufficient.^12^ Further, it could be investigated to what extent the strong family support available in many LMICs may serve as a protective factor that could influence how digital interventions are adopted and integrated into existing care structures.^69^ Qualitative investigations including both older adults and family members would aid in better understanding how these interventions are optimally developed and implemented in the context of LMIC countries. Related to this, user-centred design when developing digital interventions for older adults should be encouraged as digital literacy may vary.^70^ Second, all four studies with quantitative data focused on loneliness or depression. Although these conditions are prevalent among older adults, research actually suggests that anxiety is more common among older adults.^71^ Thus, future studies should not only focus on depression, but also on anxiety and other common mental health problems. Third, digital interventions in general are usually more effective when delivered with some form of human interaction, but it is not known whether human interaction is more (or less) important when the intervention is delivered to older adults. Further, human interaction always involves a cost that should be estimated in relation to the intervention’s effectiveness. Future research could experimentally investigate whether human interaction is beneficial for effects, and this should also be related to the specific cultural context in which it is explored. It can be conceived that human interaction is more important in a context where digital literacy is low (as is the case in many LMICs).^72^ Fourth, although CBT is the most commonly used therapy form in digital interventions, it was developed in a Western context and has proven difficult to implement in LMICs.^73^ Cultural adaptations when attempting to study and implement CBT in non-Western countries may be beneficial in some cases.^74^ Often, therapeutic strategies in CBT imply a norm that may look different in non-Western cultures, for example when it comes to socially accepted behaviours and views on stigma and shame. Fifth, some of the digital interventions used in HICs in the Riadi *et al* review used novel/high threshold devices, such as virtual reality, robotics, gaming consoles, etc.^53^ In LMICs, low-threshold technology such as simple mobile-first web pages, or apps that work on low-spec devices might currently be a better option to achieve high scalability as possible. Sixth, specific ethical aspects should be taken into consideration when developing digital interventions for older adults. For example, digital literacy may differ in different demographics (ie, income, rural) and geographic groups,^75^ and to achieve equity in access to, and usability of, digital interventions, this should be taken into consideration when, for example, planning focus groups or stakeholder compositions.

## Conclusion and recommendations for future research

Digital interventions for common mental health problems for older adults in LMICs hold promise, although published studies are few. Given the ongoing and impending demographic changes and growing burden of mental healthcare needs among older adults in LMICs, the development and expansion of research in this field is urgent. In line with our discussion earlier, we have the following recommendations to the research community at this early stage:

- Qualitative research, patient-oriented research and stakeholder involvement (including both older adults and family members) should be a priority as this research would be the best current aid in understanding how these interventions could fit into the lives of older adults in LMICs.
- Future studies should evaluate changes with well-validated questionnaires, preferably those specifically developed for older adults such as the GDS, so as to facilitate comparisons across studies (and countries) in the future.
- Future investigations of digital interventions should not only focus on depression and social isolation. Other common mental health problems among older adults, such as anxiety and sleep problems are almost equally common and should also be addressed.
- The specific importance of human interaction in digital interventions for older adults should be investigated experimentally, as its relevance could have major implications for scalability in LMICs.
- When using therapeutic interventions such as CBT, that have mainly been investigated in HICs, in LMICs, cultural adaptation of the therapeutic content may be a relevant consideration.

## Supplementary material

10.1136/bmjgh-2024-017836 online supplemental file 1

10.1136/bmjgh-2024-017836 online supplemental file 2

10.1136/bmjgh-2024-017836 online supplemental file 3

## Data Availability

No data are available.
