# Supplementary material for: Digital interventions for common mental health problems among older adults in low- and middle-income countries: a scoping review
Source: BMJ Glob Health. 2025 Jun 24;10(6):e017836. doi: 10.1136/bmjgh-2024-017836 (PMC12198822; doi:10.1136/bmjgh-2024-017836)
Supplement: online supplemental file 2 [file bmjgh-10-6-s002.pdf]

| Components | Concept | MeSH term | tiab term |
| --- | --- | --- | --- |
| **Patient / population** | Older people | Aged[mesh] | Aged[tiab]<br>Elderly[tiab] |
| | | Geriatrics[mesh] | Geriatric*[tiab]<br>Gerontology[tiab] |
| | | Aging[mesh] | Aging[tiab]<br>Senescence[tiab]<br>Biological Aging[tiab]<br>Aging, Biological[tiab]<br>4,367,665 |
| **Intervention** | Digital intervention | Telemedicine[mesh] | Telemedicine[tiab]<br>Tele-Referral*[tiab]<br>Tele Referral*[tiab]<br>Virtual Medicine[tiab]<br>Medicine, Virtual[tiab]<br>Tele-Intensive Care[tiab]<br>Tele Intensive Care[tiab]<br>Tele-ICU[tiab]<br>Tele ICU[tiab]<br>Mobile Health[tiab]<br>Health, Mobile[tiab]<br>mHealth[tiab]<br>Telehealth[tiab]<br>eHealth[tiab]<br>77,638 |
| **Comparator** | -- | -- | -- |
| **Outcome** | Common mental health disorders | Mental Health[mesh] | Health, Mental[tiab]<br>Mental Hygiene[tiab]<br>Hygiene, Mental[tiab] |
| | Mental health disorders | Mental Disorders[mesh] | Mental Disorder*[tiab]<br>Psychiatric Illness*[tiab]<br>Psychiatric Disease*[tiab]<br>Mental Illness*[tiab]<br>Illness, Mental[tiab]<br>Psychiatric Disorder*[tiab]<br>Behavior Disorder*[tiab]<br>Diagnosis, Psychiatric[tiab]<br>Psychiatric Diagnosis[tiab]<br>Mental Disorders, Severe[tiab]<br>Mental Disorder, Severe[tiab]<br>Severe Mental Disorder*[tiab] |
| | Anxiety problems | Anxiety Disorders[mesh] | Anxiety Disorder*[tiab]<br>Disorder, Anxiety[tiab]<br>Disorders, Anxiety[tiab]<br>Neuroses, Anxiety[tiab]<br>Anxiety Neuroses[tiab]<br>Anxiety States, Neurotic[tiab]<br>Anxiety State, Neurotic[tiab]<br>Neurotic Anxiety State*[tiab]<br>State, Neurotic Anxiety[tiab] |

| | | | |
|---|---|---|---|
| | | | States, Neurotic Anxiety[tiab] |
| | | Anxiety[mesh] | Anxiety[tiab]<br>Angst[tiab]<br>Social Anxiet*[tiab]<br>Anxieties, Social[tiab]<br>Anxiety, Social[tiab]<br>Hypervigilance[tiab]<br>Nervousness[tiab]<br>Anxiousness[tiab] |
| | Depressive disorders | Depressive Disorder[mesh] | Depressive Disorder*[tiab]<br>Disorder, Depressive[tiab]<br>Disorders, Depressive[tiab]<br>Neurosis, Depressive[tiab]<br>Depressive Neuros*[tiab]<br>Neuroses, Depressive[tiab]<br>Depression, Endogenous[tiab]<br>Depressions, Endogenous[tiab]<br>Endogenous Depression*[tiab]<br>Depressive Syndrome*[tiab]<br>Syndrome, Depressive[tiab]<br>Syndromes, Depressive[tiab]<br>Depression, Neurotic[tiab]<br>Depressions, Neurotic[tiab]<br>Neurotic Depression*[tiab]<br>Melancholia*[tiab]<br>Unipolar Depression*[tiab]<br>Depression, Unipolar[tiab]<br>Depressions, Unipolar[tiab] |
| | | Depression[mesh] | Depression[tiab]<br>Depressive Symptom*[tiab]<br>Symptom, Depressive[tiab]<br>Emotional Depression[tiab]<br>Depression, Emotional[tiab] |
| | | Loneliness[mesh] | Lonelines*[tiab]<br>Homesicknes*[tiab] |
| | Sleeping disorder | Sleep Initiation and Maintenance Disorders[mesh] | Sleep Initiation and Maintenance Disorder*[tiab]<br>Disorders of Initiating and Maintaining Sleep[tiab]<br>DIMS (Disorders of Initiating and Maintaining Sleep) [tiab]<br>Early Awakening[tiab]<br>Awakening, Early[tiab]<br>Nonorganic Insomnia[tiab]<br>Insomnia, Nonorganic[tiab]<br>Primary Insomnia[tiab]<br>Insomnia, Primary[tiab]<br>Transient Insomnia[tiab]<br>Insomnia, Transient[tiab]<br>Rebound Insomnia[tiab]<br>Insomnia, Rebound[tiab] |

| | | | |
|---|---|---|---|
| | | | Secondary Insomnia[tiab]<br>Insomnia, Secondary[tiab]<br>Sleep Initiation Dysfunction[tiab]<br>Dysfunction, Sleep Initiation[tiab]<br>Dysfunctions, Sleep Initiation[tiab]<br>Sleep Initiation Dysfunctions[tiab]<br>Sleeplessness[tiab]<br>Insomnia Disorder*[tiab]<br>Insomnia*[tiab]<br>Chronic Insomnia[tiab]<br>Insomnia, Chronic[tiab]<br>Psychophysiological Insomnia[tiab]<br>Insomnia, Psychophysiological[tiab] |
| | Substance use | Substance-Related Disorders[mesh] | Substance Related Disorder*[tiab]<br>Disorder, Substance Related[tiab]<br>Disorders, Substance Related[tiab]<br>Related Disorder, Substance[tiab]<br>Related Disorders, Substance[tiab]<br>Drug Use Disorder*[tiab]<br>Disorder, Drug Use*[tiab]<br>Organic Mental Disorders, Substance-Induced[tiab]<br>Organic Mental Disorders, Substance Induced[tiab]<br>Substance Abuse[tiab]<br>Abuse, Substance[tiab]<br>Substance Abuse*[tiab]<br>Substance Dependence[tiab]<br>Dependence, Substance[tiab]<br>Substance Addiction[tiab]<br>Addiction, Substance[tiab]<br>Chemical Dependence*[tiab]<br>Dependence, Chemical[tiab]<br>Dependences, Chemical[tiab]<br>Drug Dependence[tiab]<br>Dependence, Drug[tiab]<br>Drug Addiction[tiab]<br>Addiction, Drug[tiab]<br>Prescription Drug Abuse[tiab]<br>Abuse, Prescription Drug[tiab]<br>Drug Abuse, Prescription[tiab]<br>Substance Use*[tiab]<br>Use, Substance[tiab]<br>Drug Abuse[tiab]<br>Abuse, Drug[tiab]<br>Drug Habituation[tiab]<br>Habituation, Drug[tiab]<br>Substance Use Disorder*[tiab]<br>Disorder, Substance Use[tiab] |

| | | | |
|---|---|---|---|
| | | | 2,015,224 |
| **Settings** | Low middle income countries | | Low middle income countr*[tiab]<br>LMIC[tiab] |
| | India | India[mesh] | India[tiab]<br>Republic of India[tiab] |
| | List of LMIC countries other than India [17] | Algeria[mesh] | Algeria[tiab] |
| | | Angola[mesh] | Angola[tiab] |
| | | Bangladesh[mesh] | Bangladesh[tiab] |
| | | Benin[mesh] | Benin[tiab]<br>Republic of Benin[tiab]<br>Dahomey[tiab] |
| | | Bhutan[mesh] | Bhutan[tiab] |
| | | Bolivia[mesh] | Bolivia[tiab] |
| | | Cabo Verde[mesh] | Cabo Verde[tiab]<br>Republic of Cape Verde[tiab]<br>Cape Verde[tiab] |
| | | Cambodia[mesh] | Cambodia[tiab]<br>Khmer Republic[tiab]<br>Kampuchea[tiab] |
| | | Cameroon[mesh] | Cameroon*[tiab]<br>Republic of Cameron[tiab]<br>United Republic of Cameroon[tiab] |
| | | Comoros[mesh] | Comoros[tiab]<br>Iles Comores[tiab]<br>Comoro Islands[tiab]<br>Mayotte[tiab] |
| | | Democratic Republic of the Congo[mesh] | Democratic Republic of the Congo[tiab]<br>Congo (Kinshasa)[tiab]<br>Zaire[tiab]<br>Belgian Congo[tiab]<br>Katanga[tiab] |
| | | Cote d'Ivoire[mesh] | Cote d'Ivoire[tiab]<br>Ivory Coast[tiab]<br>Republic of Cote dilvoire[tiab] |
| | | Djibouti[mesh] | Djibouti[tiab]<br>Somaliland, French[tiab]<br>Republic of Djibouti[tiab]<br>French Somaliland[tiab] |
| | | Egypt[mesh] | Egypt[tiab]<br>Arab Republic of Egypt[tiab]<br>United Arab Republic[tiab] |
| | | El Salvador[mesh] | El Salvador[tiab] |
| | | Eswatini[mesh] | Eswatini[tiab]<br>Swaziland[tiab] |
| | | Ghana[mesh] | Ghana[tiab] |

| | | | |
|---|---|---|---|
| | | | Republic of Ghana[tiab]<br>Gold Coast[tiab] |
| | | Haiti[mesh] | Haiti[tiab] |
| | | Honduras[mesh] | Honduras[tiab] |
| | | Indonesia[mesh] | Indonesia[tiab]<br>Netherlands East Indies[tiab]<br>East Indies[tiab]<br>West Irian[tiab]<br>New Guinea, Indonesian[tiab]<br>New Guinea, West[tiab]<br>Indonesian New Guinea[tiab]<br>Irian Jaya[tiab]<br>Timor[tiab]<br>Java[tiab]<br>Bali[tiab]<br>Sumatra[tiab]<br>Celebes[tiab]<br>Sulawesi[tiab]<br>Malay Archipelago[tiab]<br>Madoera[tiab]<br>Madura[tiab] |
| | | Iran[mesh] | Iran[tiab]<br>Islamic Republic of Iran[tiab] |
| | | Kenya[mesh] | Kenya[tiab]<br>Republic of Kenya[tiab] |
| | | Micronesia[mesh] | Micronesia[tiab]<br>Johnston Island[tiab]<br>Kiribati[tiab]<br>Gilbert Islands[tiab]<br>Mariana Islands[tiab]<br>Marshall Islands[tiab]<br>Nauru[tiab]<br>Northern Mariana Islands[tiab]<br>Pacific Islands (Trust Territory)[tiab]<br>Tuvalu[tiab]<br>Ellice Islands[tiab]<br>Caroline Islands[tiab]<br>Micronesia, Federated States of[tiab] |
| | | Kyrgyzstan[mesh] | Kyrgyzstan[tiab]<br>Kirghizia[tiab]<br>Kyrgyz Republic[tiab]<br>Kirghiz SSR[tiab]<br>Kirgizstan[tiab]<br>Kirghiz S.S.R. [tiab] |
| | | Laos[mesh] | Laos[tiab] |
| | | Lebanon[mesh] | Lebanon[tiab]<br>Lebanese Republic[tiab] |
| | | Lesotho[mesh] | Lesotho[tiab]<br>Basutoland[tiab]<br>Kingdom of Lesotho[tiab] |
| | | Mauritania[mesh] | Mauritania[tiab] |

| | | | |
|---|---|---|---|
| | | Mongolia[mesh] | Mongolia[tiab] |
| | | Morocco[mesh] | Morocco[tiab]<br>Ifni[tiab] |
| | | Myanmar[mesh] | Myanma*[tiab]<br>Burma[tiab] |
| | | Nepal[mesh] | Nepal[tiab]<br>Federal Democratic Republic of Nepal[tiab] |
| | | Nicaragua[mesh] | Nicaragua[tiab] |
| | | Nigeria[mesh] | Nigeria[tiab]<br>Federal Republic of Nigeria[tiab] |
| | | Pakistan[mesh] | Pakistan[tiab]<br>Islamic Republic of Pakistan[tiab] |
| | | Papua New Guinea[mesh] | Papua New Guinea[tiab]<br>New Guinea, Papua[tiab]<br>New Guinea, East[tiab] |
| | | Philippines[mesh] | Philippines[tiab]<br>Phillipines[tiab]<br>Phillippines[tiab]<br>Philipines[tiab] |
| | | Samoa[mesh] | Samoa[tiab]<br>Samoan Islands[tiab]<br>Samoa Islands[tiab]<br>Navigator Island*[tiab] |
| | | Sao Tome and Principe[mesh] | Sao Tome and Principe[tiab] |
| | | Senegal[mesh] | Senegal[tiab]<br>Republic of Senegal[tiab] |
| | | Melanesia[mesh] | Melanesia[tiab]<br>Norfolk Island[tiab]<br>Solomon Islands[tiab]<br>British Solomon Islands[tiab] |
| | | Middle East[mesh] | Middle East[tiab]<br>West Bank[tiab]<br>Near East[tiab]<br>Gaza Strip[tiab]<br>Gaza Strip (Palestine)[tiab] |
| | | Sri Lanka[mesh] | Sri Lanka[tiab]<br>Ceylon[tiab] |
| | | Tajikistan[mesh] | Tajikistan[tiab]<br>Tadjikistan[tiab]<br>Tadzhik S.S.R. [tiab]<br>Tadzhik SSR[tiab]<br>Tadzhikistan[tiab] |
| | | Tanzania[mesh] | Tanzania[tiab]<br>United Republic of Tanzania[tiab]<br>Zanzibar[tiab]<br>Tanganyika[tiab] |
| | | Timor-Leste[mesh] | Timor-Leste[tiab]<br>Democratic Republic of Timor-Leste[tiab] |

| | | | |
|---|---|---|---|
| | | | East Timor[tiab] |
| | | Tunisia[mesh] | Tunisia[tiab] |
| | | Ukraine[mesh] | Ukraine[tiab] |
| | | Uzbekistan[mesh] | Uzbekistan[tiab]<br>Uzbek SSR[tiab]<br>Republic of Uzbekistan[tiab]<br>Uzbek S.S.R.[tiab] |
| | | Vanuatu[mesh] | Vanuatu[tiab]<br>New Hebrides[tiab] |
| | | Vietnam[mesh] | Vietnam[tiab]<br>Viet Nam[tiab]<br>Vietnam, Republic of[tiab]<br>North Vietnam[tiab] |
| | | Zimbabwe[mesh] | Zimbabwe[tiab]<br>Zimbabwe Rhodesia[tiab]<br>Southern Rhodesia[tiab]<br>Republic of Zimbabwe[tiab]<br>Rhodesia, Southern[tiab] |
| | | ((Afghanistan)<br>(Burundi)<br>(Burkina Faso)<br>(Central African Republic)<br>(Congo, Dem. Rep.) OR (Eritrea) OR (Ethiopia) OR (Gambia) OR (Guinea-Bissau) OR (Liberia) OR (Madagascar) OR (Mali) OR (Mozambique) OR (Malawi) OR (Niger) OR (Korea, Dem. People's Rep.) OR (Rwanda) OR (Sudan) OR (Sierra Leone) OR (Somalia) OR (South Sudan) OR (Syrian Arab Republic) OR (Chad) OR (Togo) OR (Uganda) OR (Yemen, Rep.) OR (Albania) OR (Argentina) OR (Armenia) OR (Azerbaijan) OR | ((Afghanistan) OR (Burundi) OR (Burkina Faso) OR (Central African Republic) OR (Congo, Dem. Rep.) OR (Eritrea) OR (Ethiopia) OR (Gambia) OR (Guinea-Bissau) OR (Liberia) OR (Madagascar) OR (Mali) OR (Mozambique) OR (Malawi) OR (Niger) OR (Korea, Dem. People's Rep.) OR (Rwanda) OR (Sudan) OR (Sierra Leone) OR (Somalia) OR (South Sudan) OR (Syrian Arab Republic) OR (Chad) OR (Togo) OR (Uganda) OR (Yemen, Rep.) OR (Albania) OR (Argentina) OR (Armenia) OR (Azerbaijan) OR (Bulgaria) OR (Bosnia and Herzegovina) OR (Belarus) OR (Belize) OR (Brazil) OR (Botswana) OR (China) OR (Colombia) OR (Costa Rica) OR (Cuba) OR (Dominica) OR (Dominican Republic) OR (Ecuador) OR (Fiji) OR (Gabon) OR (Georgia) OR (Equatorial Guinea) OR (Grenada) OR (Guatemala) OR (Indonesia) OR (Iraq) OR (Jamaica) OR (Kazakhstan) OR (Libya) OR (St. Lucia) OR (Moldova) OR |

| | | | |
|---|---|---|---|
| | | (Bulgaria) OR (Bosnia and Herzegovina) OR (Belarus) OR (Belize) OR (Brazil) OR (Botswana) OR (China) OR (Colombia) OR (Costa Rica) OR (Cuba) OR (Dominica) OR (Dominican Republic) OR (Ecuador) OR (Fiji) OR (Gabon) OR (Georgia) OR (Equatorial Guinea) OR (Grenada) OR (Guatemala) OR (Indonesia) OR (Iraq) OR (Jamaica) OR (Kazakhstan) OR (Libya) OR (St. Lucia) OR (Moldova) OR (Maldives) OR (Mexico) OR (Marshall Islands) OR (North Macedonia) OR (Montenegro) OR (Mauritius) OR (Malaysia) OR (Namibia) OR (Peru) OR (Palau) OR (Paraguay) OR (West Bank and Gaza) OR (Russian Federation) OR (El Salvador) OR (Serbia) OR (Suriname) OR (Thailand) OR (Turkmenistan) OR (Tonga) OR (Türkiye) OR (Tuvalu) OR (St. Vincent and the Grenadines) OR | (Maldives) OR (Mexico) OR (Marshall Islands) OR (North Macedonia) OR (Montenegro) OR (Mauritius) OR (Malaysia) OR (Namibia) OR (Peru) OR (Palau) OR (Paraguay) OR (West Bank and Gaza) OR (Russian Federation) OR (El Salvador) OR (Serbia) OR (Suriname) OR (Thailand) OR (Turkmenistan) OR (Tonga) OR (Türkiye) OR (Tuvalu) OR (St. Vincent and the Grenadines) OR (Kosovo) OR (South Africa) OR (Venezuela, RB)) |

| | | | |
|---|---|---|---|
| | | (Kosovo) OR (South Africa) OR (Venezuela, RB)) | |
