# Supplementary material for: Digital interventions for common mental health problems among older adults in low- and middle-income countries: a scoping review
Source: BMJ Glob Health. 2025 Jun 24;10(6):e017836. doi: 10.1136/bmjgh-2024-017836 (PMC12198822; doi:10.1136/bmjgh-2024-017836)
Supplement: online supplemental file 3 [file bmjgh-10-6-s003.pdf]

Scopus database:

P	((aged) OR (elderly) OR (geriatric*) OR (gerontology) OR (aging) OR (senescence) OR (biological AND aging) OR (aging, AND biological))	7,431,104
I	((telemedicine) OR (tele-referral*) OR (tele AND referral*) OR (virtual AND medicine) OR (medicine, AND virtual) OR (tele-intensive AND care) OR (tele AND intensive AND care) OR tele-icu OR (tele AND icu) OR (mobile AND health) OR (health, AND mobile) OR (mhealth) OR telehealth OR (ehealth))	184,895
O	((health, AND mental) OR (mental AND hygiene) OR (hygiene, AND mental) OR (mental AND disorder*) OR (psychiatric AND illness*) OR (psychiatric AND disease*) OR (mental AND illness*) OR (illness, AND mental) OR (psychiatric AND disorder*) OR (behavior AND disorder*) OR (diagnosis, AND psychiatric) OR (psychiatric AND diagnosis) OR (mental AND disorders, AND severe) OR (mental AND disorder, AND severe) OR (severe AND mental AND disorder*) OR (anxiety AND disorder*) OR (disorder, AND anxiety) OR (disorders, AND anxiety) OR (neuroses, AND anxiety) OR (anxiety AND neuroses) OR (anxiety AND states, AND neurotic) OR (anxiety AND state, AND neurotic) OR (neurotic AND anxiety AND state*) OR (state, AND neurotic AND anxiety) OR (states, AND neurotic AND anxiety) OR anxiety OR angst OR (social AND anxiet*) OR (anxieties, AND social) OR (anxiety, AND social) OR hypervigilance OR nervousness OR anxiousness OR (depressive AND disorder*) OR (disorder, AND depressive) OR (disorders, AND depressive) OR (neurosis, AND depressive) OR (depressive AND neuros*) OR (neuroses, AND depressive) OR (depression, AND endogenous) OR (depressions, AND endogenous) OR (endogenous AND depression*) OR (depressive AND syndrome*) OR (syndrome, AND depressive) OR (syndromes, AND depressive) OR (depression, AND neurotic) OR (depressions, AND neurotic) OR (neurotic AND depression*) OR melancholia* OR (unipolar AND depression*) OR (depression, AND unipolar) OR (depressions, AND unipolar) OR depression OR (depressive AND symptom*) OR (symptom, AND depressive) OR (emotional AND depression) OR (depression, AND emotional) OR lonelines* OR homesicknes* OR (sleep AND initiation AND maintenance AND disorder*) OR (disorders AND of AND initiating AND maintaining AND sleep) OR dims OR (early AND awakening) OR (awakening, AND early) OR (nonorganic AND insomnia) OR (insomnia, AND nonorganic) OR (primary AND	3,976,922

	insomnia) OR (insomnia, AND primary) OR (transient AND insomnia) OR (insomnia, AND transient) OR (rebound AND insomnia) OR (insomnia, AND rebound) OR (secondary AND insomnia) OR (insomnia, AND secondary) OR (sleep AND initiation AND dysfunction) OR (dysfunction, AND sleep AND initiation) OR (dysfunctions, AND sleep AND initiation) OR (sleep AND initiation AND dysfunction*) OR sleeplessness OR (insomnia AND disorder*) OR insomnia* OR (chronic AND insomnia) OR (insomnia, AND chronic) OR (psychophysiological AND insomnia) OR (insomnia, AND psychophysiological) OR (substance AND related AND disorder*) OR (disorder, AND substance AND related) OR (disorders, AND substance AND related) OR (related AND disorder, AND substance) OR (related AND disorders, AND substance) OR (drug AND use AND disorder*) OR (disorder, AND drug AND use*) OR (organic AND mental AND disorders, AND substance-induced) OR (organic AND mental AND disorders, AND substance AND induced) OR (substance AND abuse) OR (abuse, AND substance) OR (substance AND abuse*) OR (substance AND dependence) OR (dependence, AND substance) OR (substance AND addiction) OR (addiction, AND substance) OR (chemical AND dependence*) OR (dependence, AND chemical) OR (dependences, AND chemical) OR (drug AND dependence) OR (dependence, AND drug) OR (drug AND addiction) OR (addiction, AND drug) OR (prescription AND drug AND abuse) OR (abuse, AND prescription AND drug) OR (drug AND abuse, AND prescription) OR (substance AND use*) OR (use, AND substance) OR (drug AND abuse) OR (abuse, AND drug) OR (drug AND habituation) OR (habituation, AND drug) OR (substance AND use AND disorder*) OR (disorder, AND substance AND use))	
S	((India) OR (Republic of India) OR (Algeria) OR (Angola) OR (Bangladesh) OR (Benin) OR (Republic of Benin) OR (Dahomey) OR (Bhutan) OR Bolivia OR (Cabo Verde) OR (Republic of Cape Verde) OR (Cape Verde) OR Cambodia OR (Khmer Republic) OR Kampuchea OR Cameroon* OR (Republic of Cameron) OR (United Republic of Cameroon) OR Comoros OR (Iles Comores) OR (Comoro Island*) OR Mayotte OR (Democratic Republic of the Congo) OR Congo OR Zaire OR (Belgian Congo) OR Katanga OR (Cote d'Ivoire) OR (Ivory Coast) OR (Republic of Cote diIvoire) OR Djibouti OR (Somaliland, French) OR (Republic of Djibouti) OR (French Somaliland) OR Egypt OR (Arab Republic of Egypt) OR (United Arab Republic) OR (El Salvador) OR Eswatini OR Swaziland OR Ghana OR (Republic of Ghana) OR (Gold Coast) OR Haiti OR Honduras OR Indonesia OR (Netherlands East	5,584,501

	Indies) OR (East Indies) OR (West Irian) OR (New Guinea, Indonesian) OR (New Guinea, West) OR (Indonesian New Guinea) OR (Irian Jaya) OR Timor OR Java OR Bali OR Sumatra OR Celebes OR Sulawesi OR (Malay Archipelago) OR Madoera OR Madura OR Iran OR (Islamic Republic of Iran) OR Kenya OR (Republic of Kenya) OR Micronesia OR (Johnston Island) OR Kiribati OR (Gilbert Islands) OR (Mariana Islands) OR (Marshall Islands) OR Nauru OR (Northern Mariana Islands) OR (Pacific Islands) OR Tuvalu OR (Ellice Islands) OR (Caroline Islands) OR (Micronesia, Federated States of) OR Kyrgyzstan OR Kirghizia OR (Kyrgyz Republic) OR (Kirghiz SSR) OR Kirgizstan OR (Kirghiz S.S.R.) OR Laos OR Lebanon OR (Lebanese Republic) OR Lesotho OR Basutoland OR (Kingdom of Lesotho) OR Mauritania OR Mongolia OR Morocco OR Ifni OR Myanma* OR Burma OR Nepal OR (Federal Democratic Republic of Nepal) OR Nicaragua OR Nigeria OR (Federal Republic of Nigeria) OR Pakistan OR (Islamic Republic of Pakistan) OR (Papua New Guinea) OR (New Guinea, Papua) OR (New Guinea, East) OR Philippines OR Phillipines OR Phillippines OR Philipines OR Samoa OR (Samoan Islands) OR (Samoa Islands) OR (Navigator Island*) OR (Sao Tome and Principe) OR Senegal OR (Republic of Senegal) OR Melanesia OR (Norfolk Island) OR (Solomon Islands) OR (British Solomon Islands) OR (Middle East) OR (West Bank) OR (Near East) OR (Gaza Strip) OR Palestine OR (Sri Lanka) OR Ceylon OR Tajikistan OR Tadjikistan OR (Tadzhik S.S.R.) OR (Tadzhik SSR) OR Tadzhikistan OR Tanzania OR (United Republic of Tanzania) OR Zanzibar OR Tanganyika OR (Timor-Leste) OR (Democratic Republic of Timor-Leste) OR (East Timor) OR Tunisia OR Ukraine OR Uzbekistan OR (Uzbek SSR) OR (Republic of Uzbekistan) OR (Uzbek S.S.R.) OR Vanuatu OR (New Hebrides) OR Vietnam OR (Viet Nam) OR (Vietnam, Republic of) OR (North Vietnam) OR Zimbabwe OR (Zimbabwe Rhodesia) OR (Southern Rhodesia) OR (Republic of Zimbabwe) OR (Rhodesia, Southern) OR (Afghanistan) OR (Burundi) OR (Burkina Faso) OR (Central African Republic) OR (Congo, Dem. Rep.) OR (Eritrea) OR (Ethiopia) OR (Gambia) OR (Guinea-Bissau) OR (Liberia) OR (Madagascar) OR (Mali) OR (Mozambique) OR (Malawi) OR (Niger) OR (Korea, Dem. People's Rep.) OR (Rwanda) OR (Sudan) OR (Sierra Leone) OR (Somalia) OR (South Sudan) OR (Syrian Arab Republic) OR (Chad) OR (Togo) OR (Uganda) OR (Yemen, Rep.) OR (Albania) OR (Argentina) OR (Armenia) OR (Azerbaijan) OR (Bulgaria) OR (Bosnia and Herzegovina) OR (Belarus) OR (Belize) OR (Brazil) OR (Botswana) OR (China) OR (Colombia) OR (Costa Rica) OR (Cuba) OR (Dominica) OR (Dominican Republic) OR (Ecuador)	

	OR (Fiji) OR (Gabon) OR (Georgia) OR (Equatorial Guinea) OR (Grenada) OR (Guatemala) OR (Indonesia) OR (Iraq) OR (Jamaica) OR (Kazakhstan) OR (Libya) OR (St. Lucia) OR (Moldova) OR (Maldives) OR (Mexico) OR (Marshall Islands) OR (North Macedonia) OR (Montenegro) OR (Mauritius) OR (Malaysia) OR (Namibia) OR (Peru) OR (Palau) OR (Paraguay) OR (West Bank and Gaza) OR (Russian Federation) OR (El Salvador) OR (Serbia) OR (Suriname) OR (Thailand) OR (Turkmenistan) OR (Tonga) OR (Türkiye) OR (Tuvalu) OR (St. Vincent and the Grenadines) OR (Kosovo) OR (South Africa) OR (Venezuela, RB))	
	P AND I AND O AND S	592
